# Soda-Lime and Borosilicate Glass Waste as Precursors for Sustainable Geopolymers

**DOI:** 10.1021/acsomega.5c11890

**Published:** 2026-04-16

**Authors:** Caroline D. Prates, Wladmir T. Silva, Athos Silva Lima, Ana Gabriela D. Santana, Luádiny V. L. Santos, Ana Paula C. Teixeira

**Affiliations:** 28114 UFMG, Departamento de Química/Icex, Belo Horizonte, Minas Gerais, Brazil, 31270-901

## Abstract

This study reports the synthesis and characterization of geopolymers produced by using borosilicate and soda-lime glass wastes as partial substitutes for conventional geopolymer raw materials. The glass wastes were characterized by XRF, XRD, FTIR, and SEM/EDS, revealing high silica content and predominantly amorphous structures, indicating their suitability for geopolymer applications. Geopolymer formulations were prepared by partially replacing sodium silicate and metakaolin with glass incorporation levels ranging from 14% to 25%. Compressive strength tests conducted after 7 days of curing showed that soda-lime glass-based geopolymers achieved compressive strengths of approximately 30 MPa at 14% glass incorporation, while borosilicate glass-based systems reached values of up to 32 MPa and maintained strengths comparable to that of the reference matrix at higher replacement levels. XRF, XRD, FTIR, and SEM/EDS analyses confirmed the formation of geopolymeric networks and consistent chemical compositions across the systems. Overall, the results indicate that waste glass can be effectively incorporated into geopolymer formulations under ambient curing conditions, enabling the partial replacement of conventional precursors and providing a comparative assessment of the influence of different glass types on mechanical resistance.

## Graphical abstract

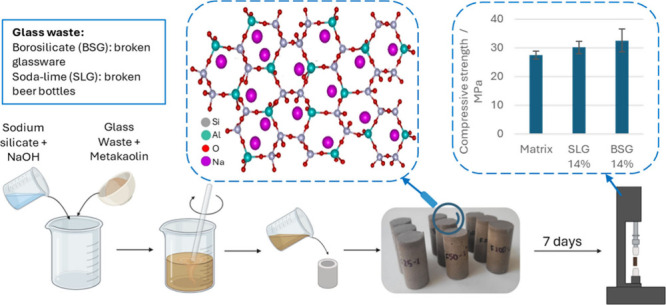

## Introduction

Geopolymers are inorganic materials formed by chemical reactions between silicates and aluminates in an alkaline medium. Due to their characteristics, geopolymers have been used in various industries, such as civil engineering, aeronautics, automobiles, and the plastics industry, among others.^1–3^

Among these applications, the one that has stood out the most is in civil construction, especially as a replacement for Portland cement. The production of Portland cement is responsible for around 8% of global CO_2_ emissions. Since geopolymers can replace cement in various applications, their use reduces the dependence on this material and, therefore, the associated emissions.^4,5^

Several types of industrial wastes and residues rich in silica and alumina have been studied as raw materials to produce geopolymers. These residues offer advantages because they are abundant and often underutilized, can reduce the amount of materials disposed of in landfills, and reduce the need to extract new raw materials. Among the main wastes studied, mining waste stands out, which has been generated on a large scale and requires alternative uses.^6–11^

To produce geopolymers, the conventional raw materials include metakaolin and an activating solution composed of sodium or potassium silicate and sodium or potassium hydroxide. Among these materials, the activating solution, particularly sodium or potassium silicates, has a high cost, which may render large-scale geopolymer production as a substitute for Portland cement economically unfeasible. Therefore, the search for low-cost materials that can partially replace silicates is highly beneficial to ensure the economic viability of geopolymer production. Many studies in the literature involve the use of tailings, particularly those rich in crystalline silica (such as mining tailings with high quartz content). However, to potentially replace silicates, a material rich in amorphous and reactive silicon is required, as is the case with glass.^6,12,13^

It is estimated that approximately 100 million tons of glass waste is generated globally each year. Despite being a fully recyclable material, a substantial portion of this waste is still disposed of in landfills, representing a significant environmental challenge. Globally, only about 21% of the glass produced is recycled, which is notably low considering its complete recyclability.^14,15^ In Brazil, according to Circula Vidro, the national entity responsible for managing the reverse logistics of glass packaging, the glass packaging industry achieved a recycling rate of 25% in 2024, meeting the target established by the Ministry of the Environment and Climate Change. Of the 877 thousand tons of glass consumed, approximately 221 thousand tons were returned to the industry for reprocessing.^15,16^

Although the glass industry has consolidated technologies, there is still a large environmental liability to be absorbed, making the development of new applications essential.^17,18^ In the construction sector, studies indicate that the incorporation of vitreous waste in the production of concrete, mortar, and cementitious materials improves their mechanical properties and contributes to the reduction of the consumption of natural raw materials.^14,18–20^

In addition to its incorporation in conventional cementitious materials, recent studies have explored alternative pathways for the valorization of waste glass in alkali-activated and geopolymeric systems.^21,22^ The performance of these materials is strongly influenced by the physicochemical characteristics of the glass, particularly its chemical composition, amorphous structure, and particle size distribution.^23^ Finely ground glass powders tend to exhibit higher reactivity in alkaline environments due to their larger specific surface area, which enhances dissolution and participation in geopolymerization reactions.^24^ Moreover, variations in glass composition, such as differences in Na_2_O, CaO, or B_2_O_3_ content, can significantly affect the reaction kinetics, gel structure formation, and resulting mechanical properties of the materials.^25^ Consequently, understanding how different types of glass waste behave in geopolymer matrices has become an important topic in recent research aimed at developing more efficient and sustainable alkali-activated binders.^14,26^

Generally, glass can act as a partial precursor, aggregate, source of reactive silica, or component of the alkaline activator, directly influencing the composition and microstructure of the material.^27^ Siddika et al. provided a comprehensive review on the use of waste glass in both Portland cement and geopolymer concretes, highlighting the participation of amorphous silica in hydration and geopolymerization reactions, refinement of the microstructure, and enhanced durability, provided that particle size and content are controlled.^14^ Cendrowski et al. incorporated ground laboratory glass waste into fly ash-based geopolymers, reporting improved microstructural features and optimized performance at intermediate replacement levels.^28^ Kotsay and Grabowski partially replaced metakaolin with soda-lime waste glass (10–50%), observing increased paste workability and the formation of a denser, more continuous geopolymeric gel at moderate contents.^29^ Tho-In et al. used waste glass powder as a partial substitute for high-calcium fly ash, showing that glass with higher CaO content promoted the formation of additional C–S–H and C–A–S–H phases, contributing to a more compact microstructure.^19^ Torres-Carrasco and Puertas employed milled glass as part of the alkaline activator in fly ash-based systems, demonstrating that the waste could serve as an effective silicon source and partially replace conventional NaOH solutions.^20^ Complementarily, Vinai and Soutsos produced sodium silicate powder from recycled glass cullet, demonstrating the feasibility of fully substituting commercial sodium silicate, thereby reducing costs and the environmental impact.^30^

Collectively, these studies highlight the versatility of waste glass in geopolymers, showing that its effect depends on the chemical composition, matrix type, and activation and curing conditions and that it can function effectively both as a precursor and as an activator in the synthesis of sustainable alternative binders.

Despite the observed advancements, the literature still presents significant gaps regarding the utilization of different types of waste glass and their specific role in the activation chemistry of geopolymers. Most studies focus on the use of soda-lime glass, primarily exploring it as a supplementary addition or partial substitute for solid precursors, whereas investigations involving borosilicate glasses—widely available in laboratories and industries—remain scarce. Furthermore, few studies systematically examine the substitution of commercial sodium silicate with glass waste.^14,19,20,27–32^ In this context, comparing different types of glass and evaluating their potential as alternative sources of silicon and sodium represent scientifically relevant opportunities for the development of more sustainable and economically viable geopolymers.

## Experimental Section

To produce geopolymers, glasses with two different compositions were tested: soda-lime glass (SLG) and borosilicate glass (BSG). The BSG was obtained from broken glassware from the Chemistry Department of UFMG, while the SLG was obtained from old beer bottles. Both glass samples were pulverized in a ball mill to a particle size smaller than 120 mesh. The glasses were characterized by X-ray diffraction (XRD), X-ray fluorescence (XRF), infrared spectroscopy (FTIR), and scanning electron microscopy (SEM/EDS).

The geopolymer formulations were prepared by partially replacing conventional raw materials with glass powder at substitution ratios of 14, 17.4, 21.5, and 25 wt %. The compositions of the prepared geopolymers are summarized in Table 1. The samples were designated according to the type and amount of glass incorporated: SLG refers to soda-lime glass, and BSG refers to borosilicate glass, followed by a numerical value indicating the percentage of glass added (e.g., SLG14 and BSG14 correspond to 14% glass incorporation). A conventional matrix was produced without the addition of glass, and based on the elemental composition of the glass residues, theoretical proportions were calculated to partially replace conventional raw materials with glass waste. This substitution was designed primarily to replace sodium silicate while ensuring comparable Si, Al, and Na contents in the SLG14 and BSG14 formulations, with minor adjustments applied to the remaining compositions. The standard raw materials used were commercial metakaolin (Metacaulim do Brasil), sodium silicate (Sulfal Química), and sodium hydroxide (Sulfal Química).

**Table 1:** Composition of the Geopolymers Produced

Nomenclature	Glass type	Glass/%	Metakaolin/%	Sodium silicate/%	Sodium hydroxide/%
Matrix	-	0.0	54.1	40.5	5.4
SLG14	SLG	14.0	57.5	21.0	7.5
SLG17.4	SLG	17.4	54.1	21.0	7.5
SLG21.5	SLG	21.5	50.0	21.0	7.5
SLG25	SLG	25.0	46.5	21.0	7.5
BSG14	BSG	14.0	57.5	21.0	7.5
BSG17.4	BSG	17.4	54.1	21.0	7.5
BSG21.5	BSG	21.5	50.0	21.0	7.5
BSG25	BSG	25.0	46.5	21.0	7.5

To prepare the materials, the components were added according to Table 1, and water was added until a pasty mixture was obtained. The materials were mixed until completely homogenized and were placed in small cylindrical molds (2 cm in diameter and 4 cm in height) with the aid of a rod and vibration. The samples were cured under ambient laboratory conditions on the laboratory bench at an approximate temperature of 25 ± 2 °C and relative humidity of approximately 50–60%. The materials were demolded after 24 h of curing. Mechanical resistance tests to compression were performed after 7 days of curing. The tests were performed in triplicate; for each composition, 3 specimens were made to perform the resistance tests. In addition to mechanical strength tests, the geopolymers produced were characterized by the following techniques: X-ray diffraction (XRD), X-ray fluorescence (XRF), infrared spectroscopy (FTIR), and scanning electron microscopy (SEM/EDS).

X-ray fluorescence (XRF) analyses were performed at the School of Mining Engineering, UFMG, using an energy-dispersive XRF spectrometer (ARL QUANT’X, Thermo Scientific) with a silver anode (Ag) X-ray tube (50 W) under vacuum; data acquisition was carried out with WINTRACE software, and spectra were processed using Uniquant software based on fundamental parameters. X-ray diffraction (XRD) analyses of powdered samples (<150 μm) were conducted at the School of Mining Engineering, UFMG, using a Philips-PANalytical PW3710 diffractometer with Cu Kα radiation and a graphite monochromator over a 2θ range of 3–90°, step size of 0.02° 2θ, and counting time of 3 s per step, with peak identification performed using Search Match software. FTIR analyses were performed at the Department of Chemistry, UFMG, using a Bruker Alpha II spectrometer in ATR mode, covering the spectral range of 4000 to 550 cm^–1^, with a resolution of 4 cm^–1^ and 16 scans per sample. Scanning electron microscopy (SEM) and energy-dispersive spectroscopy (EDS) analyses were carried out at the Microscopy Center, UFMG, using an FEI Quanta 200 FEG microscope equipped with a field emission gun (FEG); SEM images were acquired at 5 kV with a spot size of 4.5 and a 30 μm aperture, while EDS measurements were performed at 15 kV with a 50 μm aperture. Compressive strength tests were conducted at the School of Mining Engineering, UFMG, using a Contenco hydraulic press with a 20 ton load cell, applying a loading rate of 0.2–0.3 MPa/s.

## Results and Discussion

Borosilicate glass (BSG) is a material that stands out for its thermal and chemical resistance, in addition to being very durable. It is composed of silicon oxide and boron oxide and is also known as refractory glass.^33^ The glass was obtained from broken glassware from the Chemistry Department of UFMG. Soda-lime glass (SLG) is a type of glass that contains sodium oxide and calcium oxide, and it is the most common and used in several applications: glasses and plates, packaging, and household items.^29^ It was obtained from old beer bottles. Figure S1 shows the glass samples used.

### Characterization of Glass Waste Used

Table 2 shows the chemical compositions of the main elements in the glass samples obtained by X-ray fluorescence (XRF) analysis. For borosilicate glass, the composition is mainly made up of silicon and aluminum. Boron was not detected by X-ray fluorescence (XRF) due to its low atomic number and the limitations of the technique.^34^ Compared to typical borosilicate glasses reported in the literature, which generally contain 60–74% SiO_2_, 7–17% Al_2_O_3_, 9–25% B_2_O_3_, and 2–7% Na_2_O, the main differences are the higher Al_2_O_3_ content and the apparent absence of B_2_O_3_ in the XRF data.^33,35–38^ The elevated Al_2_O_3_ may be related to the specific source of the glass, consisting of laboratory glassware, often enriched in alumina to improve chemical resistance and thermal stability.^39,40^ Although boron could not be quantified, its presence is assumed as a fundamental constituent of borosilicate glass.

**Table 2:** Analysis of the Mineral Phases Present in the Glasses

Mineral phase content/%	Soda-lime glass	Borosilicate glass
SiO_2_	66.6	56.7
Na_2_O	16.0	3.3
CaO	10.9	-
Al_2_O_3_	2.0	37.9
MgO	1.9	-

The soda-lime glass used in this study exhibited a composition made up mainly of silicon, sodium, and calcium. This is generally consistent with the typical composition of soda-lime glasses reported in the literature, which usually contains approximately 70–75% SiO_2_, 12–16% Na_2_O, 10–15% CaO, 1–2% Al_2_O_3_, and 4–6% MgO. The slightly lower MgO content and slightly higher SiO_2_ observed in the current sample may be related to the specific origin of the glass, consisting of used beverage bottles.^20,27^

The X-ray diffraction patterns of both glass samples exhibited the typical characteristics of amorphous materials, with the absence of sharp diffraction peaks and the presence of a broad hump in the 10–30° (2θ) range (Figure 1), confirming that both glasses are predominantly amorphous. Similar patterns have been widely reported in the literature for waste-derived and commercial glasses, including soda-lime and borosilicate compositions. The absence of crystalline phases is advantageous for geopolymerization, as the amorphous Si–O–Al network enhances the dissolution of reactive species during alkaline activation.^14,32^

**Figure 1:**
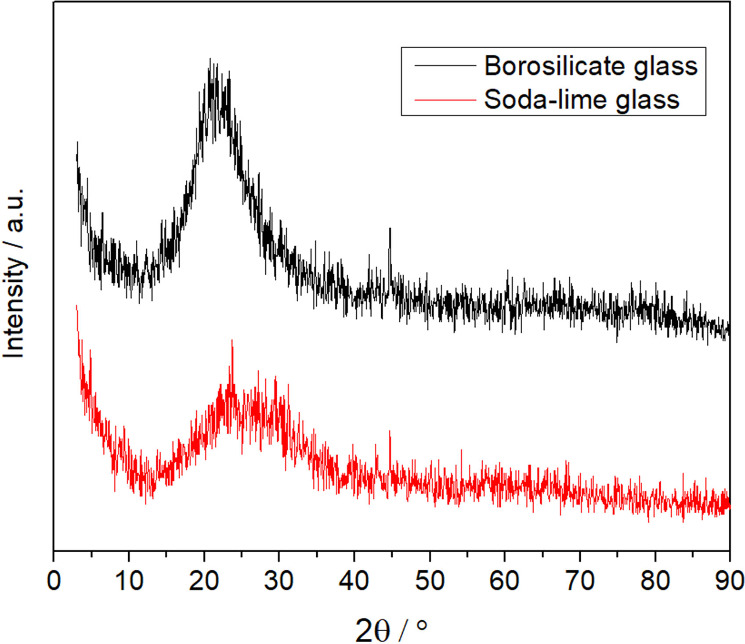
X-ray diffractograms of glasses.

Scanning electron microscopy (SEM/EDS) was employed to evaluate the morphology and elemental composition of the glass powders. Figure 2 presents the obtained micrographs and the corresponding EDS elemental maps. Both materials exhibit a heterogeneous morphology, with irregularly shaped particles and a wide range of particle sizes. Although both glasses were ground to achieve particle sizes below 120 mesh, the soda-lime glass showed larger and more heterogeneous particles compared to the borosilicate glass. This difference in particle size distribution may influence the reactivity during the geopolymerization process, as finer particles tend to dissolve more readily under alkaline conditions.^14^ Elemental mapping confirmed the compositional trends observed by XRF, indicating the predominance of silicon, sodium, and aluminum in the borosilicate glass and silicon, sodium, and calcium in the soda-lime glass.

**Figure 2:**
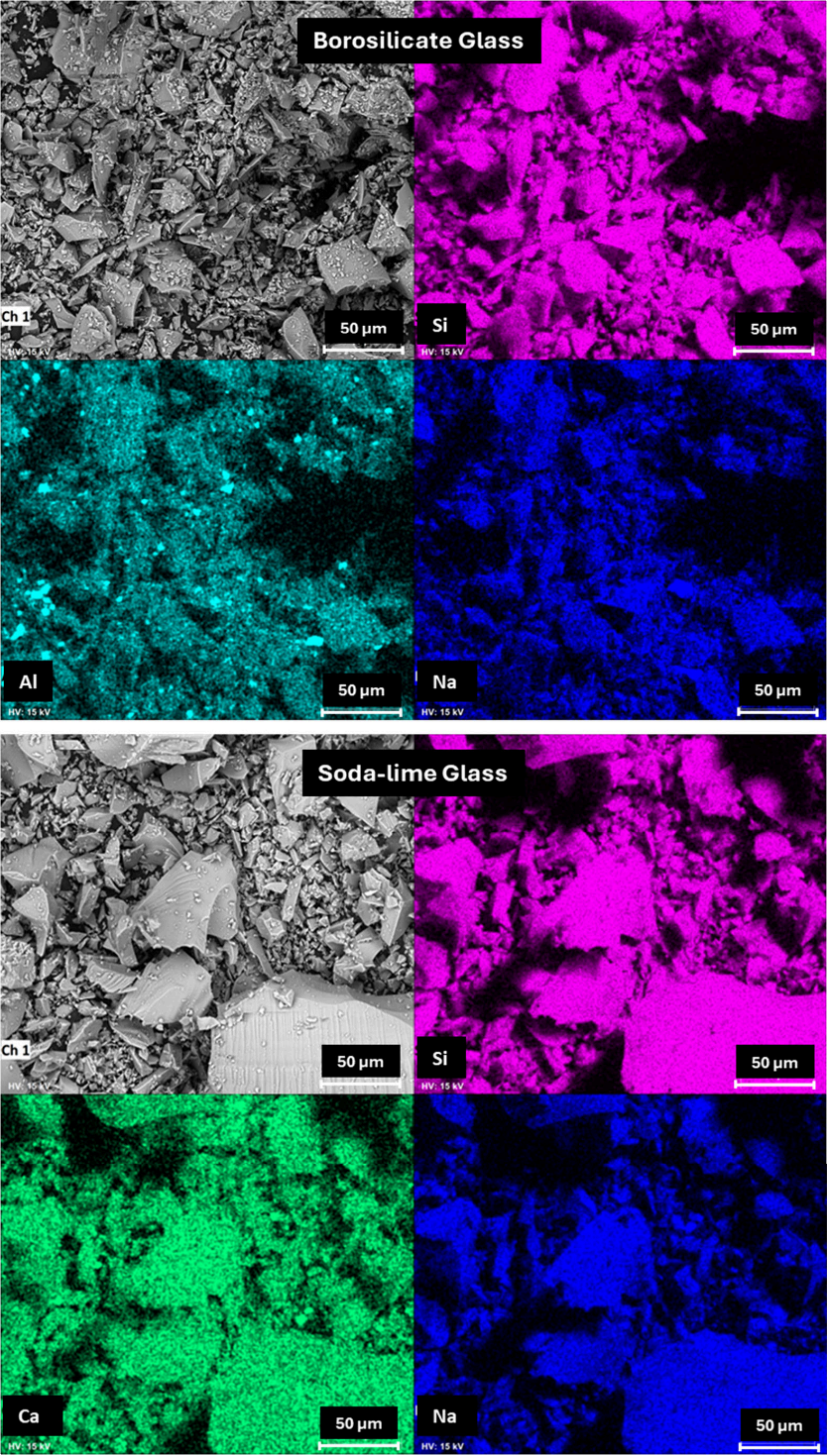
SEM images and elemental mapping of glasses.

### Geopolymer Production

The characterization results of the glass residues indicated that both materials present a predominantly amorphous structure and high silicon content, confirming their potential as suitable precursors for geopolymer synthesis. The amorphous nature of the glasses, as revealed by XRD, favors the dissolution of reactive Si–O and Al–O species under alkaline activation, while the chemical composition, rich in silicon and containing alkali elements, suggests that they can partially replace conventional raw materials used in geopolymer production.^14^ In this work, the main objective was to replace sodium silicate with glass residues while maintaining a balanced Si/Al ratio in the formulations. A reference matrix was produced using only conventional raw materials, based on previous formulations developed by the research group and consistent with those reported in the literature.^6,13^ From this standard composition, new mixtures were proposed in which a large portion of sodium silicate and a smaller fraction of metakaolin were replaced by soda-lime and borosilicate glass powders. Glass incorporation levels of 14%, 17.4%, 21.5%, and 25% were evaluated in the geopolymer formulations. The detailed compositions of all mixtures are presented in Table 1, in the Experimental Section. Figure S2 shows the specimens of the matrix, SLG14, and BSG14 geopolymers before demolding.

Compressive strength tests were performed in triplicate after 7 days of curing, and the results are shown in Figure 3. The reference matrix, produced with conventional raw materials, exhibited a compressive strength of approximately 27 MPa. For the geopolymers containing soda-lime glass, the highest strength value was obtained for the composition with 14% glass incorporation, reaching nearly 30 MPa, which is higher than that of the reference matrix.

**Figure 3:**
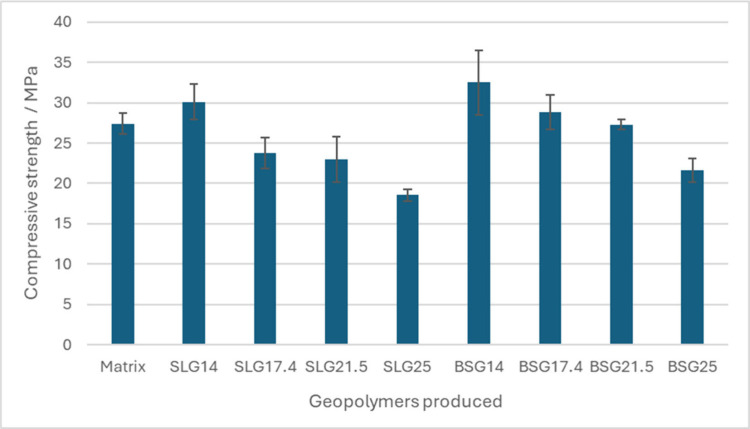
Comparative graph of the resistance values obtained for the geopolymers produced.

However, a gradual decrease in strength was observed as the glass content increased, with values dropping below 25 MPa for higher incorporation levels. The decrease in compressive strength at higher glass incorporation levels can be attributed to the replacement of highly reactive precursor materials by glass particles with lower dissolution rates under alkaline activation conditions. Although waste glass contains a large amount of amorphous silica, its dissolution is generally slower than that of metakaolin, which may limit the availability of soluble species required for geopolymer gel formation. As a result, higher glass contents may reduce the extent of geopolymerization, leading to a less dense matrix and lower mechanical strength.^14,20^ A similar trend was observed for the borosilicate glass geopolymers, in which the maximum compressive strength (around 32 MPa) was also achieved with 14% glass addition. Although the strength decreased with further increases in glass content, the formulation containing 21.5% borosilicate glass still maintained a strength comparable to the that of the reference matrix (27 MPa), suggesting that higher incorporation levels are more feasible for this type of glass.

The error bars represent the standard deviation calculated from three specimens per composition, with deviations of up to approximately ±3 MPa, which is consistent with the variability commonly reported for geopolymer systems. Although partial overlap is observed between some formulations, the trends across the different replacement levels remain consistent, with borosilicate glass-based systems tending to exhibit higher compressive strength than soda-lime glass-based systems at comparable glass contents.

Both types of glass demonstrated considerable potential as precursors for geopolymer synthesis, contributing effectively to the compressive strength of the materials. The superior performance of borosilicate glass can be attributed to its chemical composition, particle size, and particle size distribution. Scanning electron microscopy (SEM) analysis indicated that borosilicate glass consists of finer and more homogeneously distributed particles, which are likely to enhance dissolution during alkaline activation and promote the formation of a well-connected geopolymeric network. Furthermore, X-ray fluorescence (XRF) analysis revealed that borosilicate glass contains a significantly higher aluminum content, which may lead to geopolymer networks with lower Si/Al ratios. According to the literature, lower Si/Al ratios favor the development of more three-dimensional aluminosilicate frameworks, resulting in improved mechanical strength.^2^

The combination of fine particle size, homogeneous distribution, and higher aluminum content in borosilicate glass appears to enhance the geopolymerization process, resulting in denser microstructures and higher compressive strengths. These findings align with previous studies showing that both chemical composition and particle characteristics of glass precursors critically influence geopolymer performance.^14,19,28^

In addition to the particle size and aluminum content, differences in the chemical composition and network structure of the two types of glass may also contribute to the distinct mechanical behavior observed. Soda-lime glass typically contains higher amounts of CaO and Na_2_O, which can influence the geopolymerization process and the structure of the reaction products.^21,25^ In contrast, borosilicate glass contains B_2_O_3_, which modifies the glass network and may facilitate its dissolution in alkaline media, enhancing the interaction between the glass particles and the activating solution.^41^ These structural differences can favor the formation of a more homogeneous geopolymeric matrix in systems containing borosilicate glass, which may partially explain the slightly higher compressive strength values observed for these formulations.^14^

The results of compressive strength obtained in this study are in good agreement with previous literature on the incorporation of waste glass in geopolymers. Cendrowski et al. produced fly ash-based geopolymers with 0–30% ground laboratory glass, using NaOH and Na_2_SiO_3_ as activators. Glass partially replaced the solid precursor. Samples were cured at 60 °C for 48 h, reaching compressive strengths of 17.0, 41.7, 32.6, and 19.1 MPa for 0%, 10%, 20%, and 30% glass, respectively, after 28 days.^28^

Kotsay and Grabowski studied metakaolin-based pastes with 10–50% soda-lime glass replacement. Glass increased workability and allowed the reduction of liquid activator. Samples were cured between 20 and 80 °C. At 20 °C and 7 days, the compressive strengths were 32, 31, 34, and 16 MPa for 0%, 10%, 30%, and 50% glass. Higher curing temperatures and 28 day curing improved the strength, with up to 30% substitution maintaining or enhancing performance.^29^

Tho-In et al. investigated high-calcium fly ash partially replaced by fluorescent lamp (FP) and container glass (CP) at 10–40%. Samples were cured at 60 °C for 48 h and stored at 23 °C until testing. After 7 days, FP samples reached 41.1–33.3 MPa, while CP samples reached 44.4–39.9 MPa. The superior CP performance was attributed to higher CaO content (12.8%), promoting additional C–S–H and C–A–S–H phases, with maximum strength attained at 20% CP (47.6 MPa).^19^

Torres-Carrasco and Puertas evaluated waste glass as an alkaline activator in fly ash geopolymers. The systems tested included NaOH 8 M, NaOH 10 M + 15% water glass, and NaOH 10 M + 10–25 g/100 mL waste glass. Samples were cured at 85 °C for 20 h in sealed bags. Compressive strengths after 7 days ranged from 15 to 35 MPa. Glass addition provided extra silicon, modifying the N–A–S–H gel and producing a denser microstructure, demonstrating its effectiveness as an activator.^20^

Compared with the studies discussed, the geopolymers developed in this work exhibited equivalent compressive strength values. It is noteworthy that most previous studies employed more complex and costly preparation conditions, conditions that favor increased mechanical resistance, such as curing at elevated temperatures (60–85 °C) and performing compressive strength tests after 28 days. In the present study, aiming to simplify the process and reduce costs with scalability in mind, curing was performed at room temperature, and compressive strength results were obtained after only 7 days. This demonstrates the high reactivity of the materials used, particularly the glass powders, and confirms the efficiency of the adopted activation conditions. Achieving compressive strengths above 30 MPa after only 1 week represents a significant technological advantage, especially when compared to cementitious systems or other geopolymer formulations that typically require 28 days of curing to reach similar strength levels.^42^ These results highlight the potential of waste glass as partial replacements for both sodium silicate and metakaolin, contributing to the cost reduction and enhanced environmental sustainability of the process.

For the geopolymers exhibiting the best compressive strength, specifically those with 14 wt % glass incorporation (BSG14 and SLG14), as well as the reference matrix, further structural and compositional characterizations were carried out. These analyses included X-ray fluorescence (XRF) for precise elemental composition, X-ray diffraction (XRD) to assess the development of crystalline and amorphous phases, Fourier transform infrared spectroscopy (FTIR) to investigate the formation of geopolymeric networks through characteristic Si–O–Al and Si–O–Si vibrations, and scanning electron microscopy coupled with energy-dispersive X-ray spectroscopy (SEM/EDS) to examine morphology, particle distribution, and elemental mapping.

The materials were analyzed by X-ray fluorescence (XRF), and Table 3 presents the chemical composition expressed as oxides as well as the molar Si/Al and Na/Al ratios calculated from these data. The Si/Al ratios ranged from 2.59 to 3.00, which lies within the range commonly associated with the formation of well-polymerized geopolymeric networks. The BSG14 geopolymer exhibits the lowest Si/Al ratio (2.59), indicating a higher relative proportion of aluminate units in the network. According to the literature, this condition may favor a higher density of cross-linking between SiO_4_ and AlO_4_^–^ tetrahedra, potentially contributing to a stiffer matrix and slightly higher compressive strength.^2^

**Table 3:** Analysis of the Mineral Phases Present in the Geopolymers Produced

Mineral phase content/%	Matrix	BSG14	SLG14
SiO_2_	50.21	48.04	60.99
Al_2_O_3_	14.56	15.72	17.21
Na_2_O	23.35	26.01	14.66
CaO	0.47	0.55	2.04
Fe_2_O_3_	4.96	4.59	1.22
TiO_2_	2.87	2.76	1.10
Si/Al molar ratio	2.92	2.59	3.00
Na/Al molar ratio	2.64	2.73	1.40

The Na/Al ratio varied between 1.40 and 2.73, reflecting differences in the availability of alkali cations for the charge balancing of [AlO_4_]^−^ units and for the formation of the N–A–S–H gel. In the case of BSG14, the relatively high Na/Al ratio (2.73) may have promoted precursor dissolution and network formation during the early stages of geopolymerization, without clear evidence of detrimental effects on compressive strength. In contrast, the SCG14 geopolymer presents the highest Si/Al ratio (3.00) and the lowest Na/Al ratio (1.40), which may indicate a more silica-rich network and a lower availability of alkali cations, consistent with its intermediate mechanical behavior. The matrix material, with intermediate Si/Al (2.92) and relatively high Na/Al (2.64) values, may exhibit a slightly less efficient microstructure, resulting in lower compressive strength.^43,44^

In addition, the presence of boron in the BSG14 geopolymer, although not quantified by XRF, may act as a structural modifier of the aluminosilicate network, as borate species can assume tetrahedral coordination under highly alkaline conditions and partially incorporate into the geopolymeric framework.^41^ The low calcium content (∼2%) in the SLG14 geopolymer as well as the small amounts of Fe_2_O_3_ and TiO_2_ detected in all systems are expected to have a minimal influence on the microstructure and compressive strength. Overall, the observed differences in compressive strength can be attributed to the combined and subtle effects of molar ratios and chemical composition, rather than to a single dominant parameter.

The geopolymers were analyzed by X-ray diffraction (XRD) to identify the crystalline and amorphous phases formed during geopolymerization. Figure 4 presents the diffractograms obtained for the geopolymers BSG14, SLG14, and the conventional matrix, along with the diffractograms of the raw materials (glasses and metakaolin) for comparison. The diffractograms of the geopolymers reveal primarily the presence of quartz (SiO_2_, JCPDS 46-1045) as the only significant crystalline phase, consistent with the residual quartz observed in the metakaolin. No other crystalline phases were detected with notable intensity, indicating that the incorporation of glass did not lead to the formation of new unwanted crystalline compounds.

**Figure 4:**
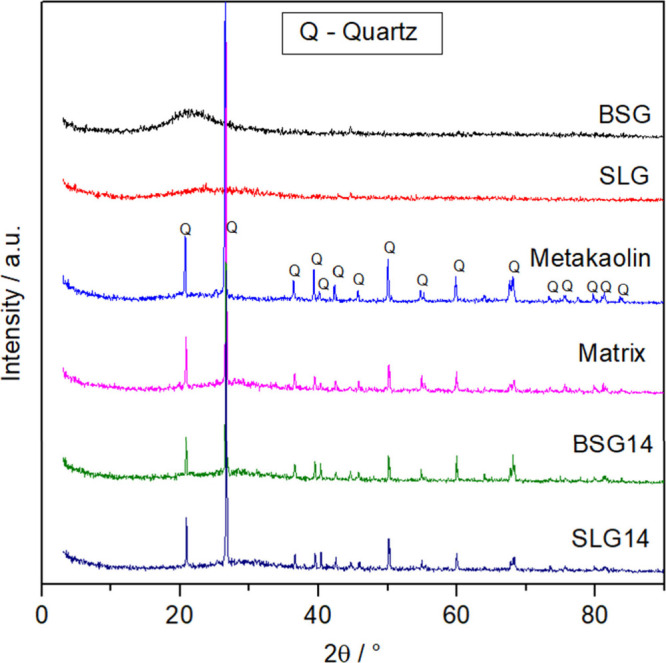
X-ray diffraction patterns of the produced geopolymers and precursor materials.

Importantly, all geopolymers exhibit a broad hump between 10° and 30° 2θ, characteristic of amorphous aluminosilicate networks, which is a hallmark of successful geopolymer formation. This amorphous hump is similar to those reported in other studies of synthesized geopolymers, where the broad diffuse peak indicates the formation of disordered three-dimensional Si–O–Al frameworks.^11,13^

Fourier transform infrared (FTIR) spectroscopy was employed to characterize the chemical structures of the raw materials (metakaolin and waste glasses) and the geopolymers produced. Figure 5 shows the spectra obtained for all of the samples. In all spectra, a prominent band around 1000 cm^–1^ is observed, corresponding to the asymmetric stretching vibrations of Si–O–Si and Si–O–Al tetrahedral bonds, which are the main building blocks of the aluminosilicate network. Notably, in the raw metakaolin and borosilicate glass samples, this band is located closer to 1000 cm^–1^, whereas in the soda-lime glass and geopolymer spectra, a shift toward lower wavenumbers occurs. The soda-lime glass has a high SiO_2_ content (66.6%) and a low Al_2_O_3_ content (2%), resulting in a network predominantly composed of Si–O–Si linkages. This predominance modifies the strength of the tetrahedral bonds compared to Si–O–Al linkages, affecting the vibrational behavior of the aluminosilicate network and causing the main FTIR band to shift toward lower wavenumbers, as widely reported for aluminosilicate systems in the literature.^45–48^ In the spectra of geopolymers, this shift is indicative of aluminum incorporation into the tetrahedral network, consistent with the formation of a three-dimensional geopolymeric structure. The magnitude of this band shift has been correlated in the literature with the degree of geopolymerization and network connectivity, confirming that both borosilicate and soda-lime glasses were effectively activated and integrated into the geopolymer matrix.^49,50^

**Figure 5:**
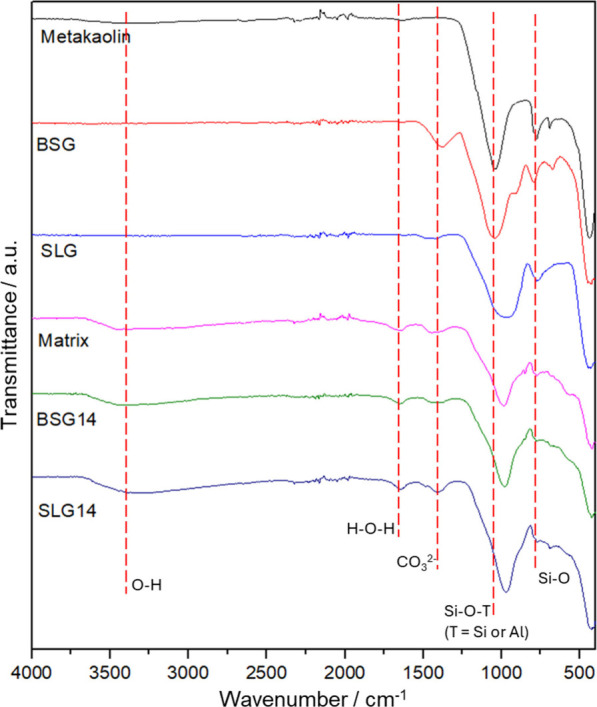
FTIR spectra of the produced geopolymers and precursor materials.

Additionally, a band around 750 cm^–1^, associated with Si–O bending vibrations, is present in all samples, reflecting structural rearrangements during geopolymerization.^51^ A band at approximately 1450 cm^–1^ is also observed in the geopolymers, corresponding to C–O stretching vibrations of carbonates, suggesting that a mild carbonation process occurred during curing.^52,53^ This feature is commonly reported in geopolymers and is associated with the natural carbonation of the material, which occurs due to the reaction of residual alkali hydroxides (Na^+^, K^+^) with atmospheric CO_2_ during handling or curing, forming carbonates such as Na_2_CO_3_ or K_2_CO_3_. Finally, broad bands at approximately 3400 and 1650 cm^–1^ indicate the presence of adsorbed water and hydroxyl groups, which are typical of geopolymeric materials and play a role in network stabilization and ion mobility.^54^

Overall, the FTIR results indicate that the geopolymerization process effectively transformed the amorphous glass and metakaolin precursors into a disordered three-dimensional aluminosilicate network, with both types of glass contributing to network formation.

The scanning electron microscopy (SEM) images and energy-dispersive X-ray spectroscopy (EDS) analyses of the geopolymers are shown in Figures 6, 7, and 8. When the SEM images are compared, it can be observed that the geopolymer produced with soda-lime glass exhibits larger and more heterogeneous particles compared to both the reference matrix and the borosilicate glass geopolymers. This morphology reduces the specific surface area available for dissolution, decreasing the reactivity during geopolymerization. Consequently, the formation of the three-dimensional aluminosilicate network is less efficient, which aligns with the lower compressive strength values observed for the soda-lime glass samples. In contrast, the borosilicate glass samples, with finer and more uniformly distributed particles, promote a denser network structure and superior mechanical performance.^14^ Elemental mapping indicates a predominant composition composed of silicon (Si), aluminum (Al), and sodium (Na), which is consistent with the X-ray fluorescence (XRF) results.

**Figure 6:**
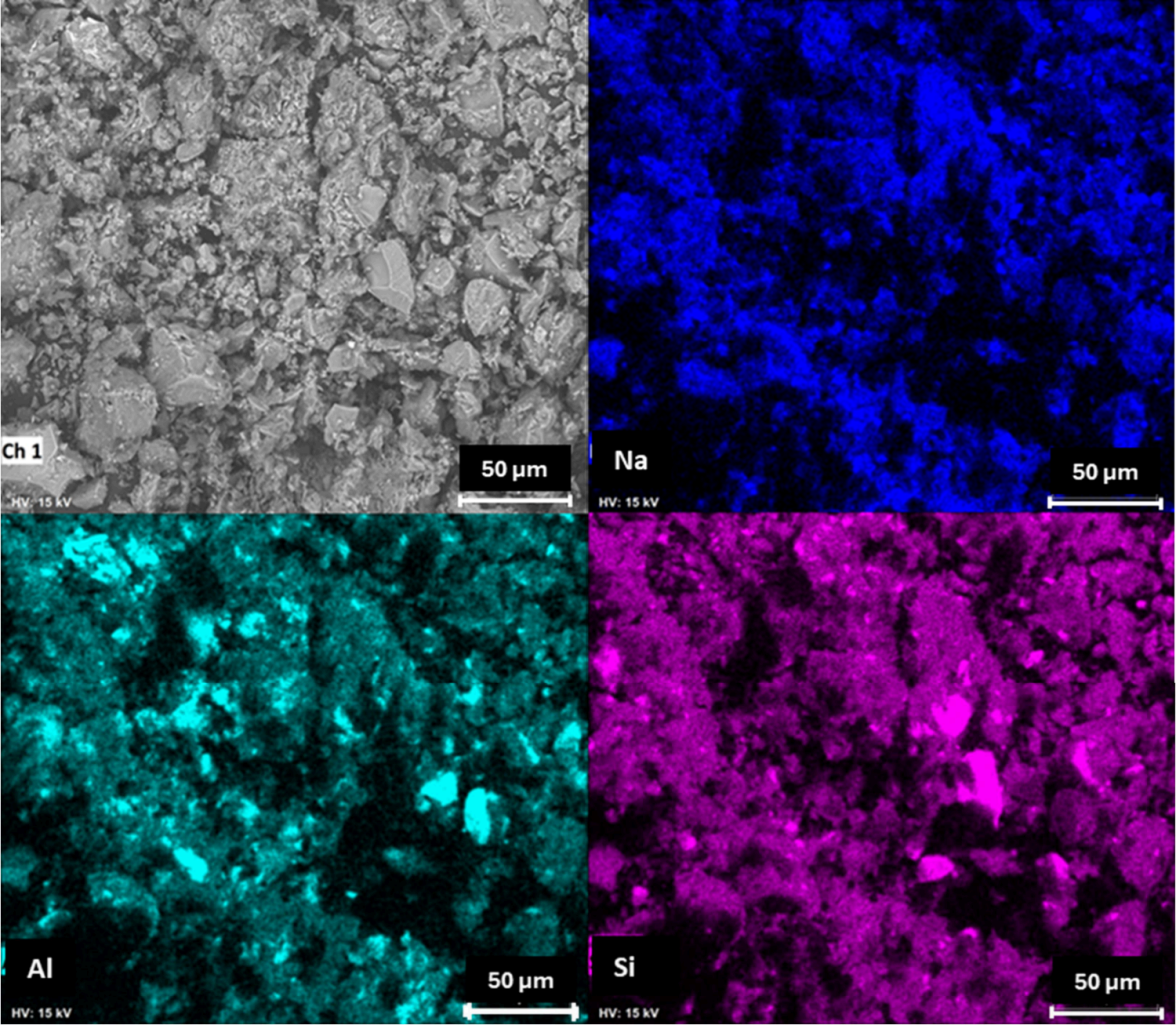
SEM image and elemental mapping of the geopolymer produced without the addition of glass.

**Figure 7:**
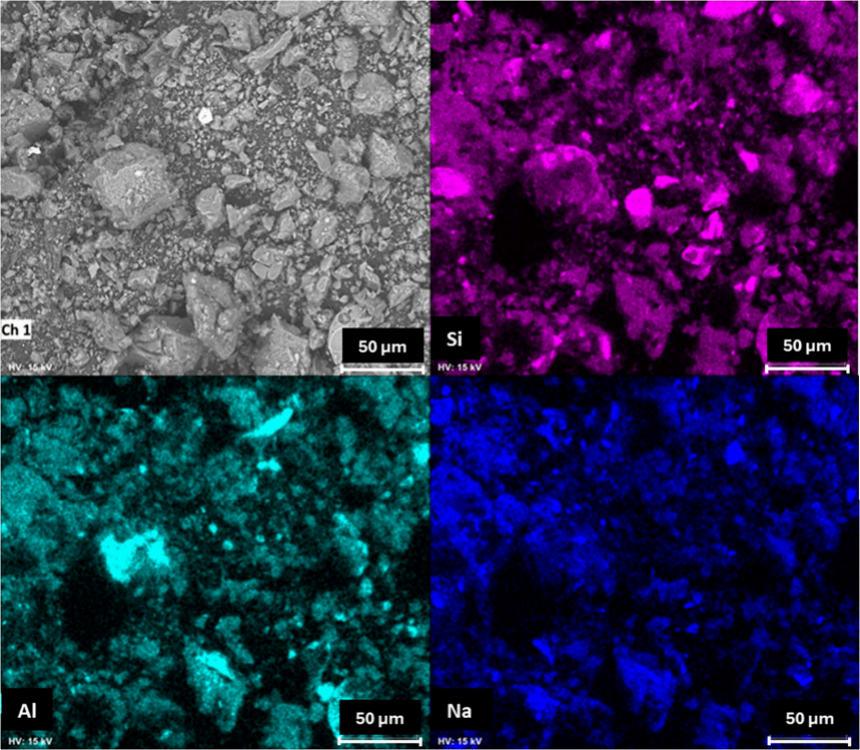
SEM image and elemental mapping of the geopolymer produced with borosilicate glass.

**Figure 8:**
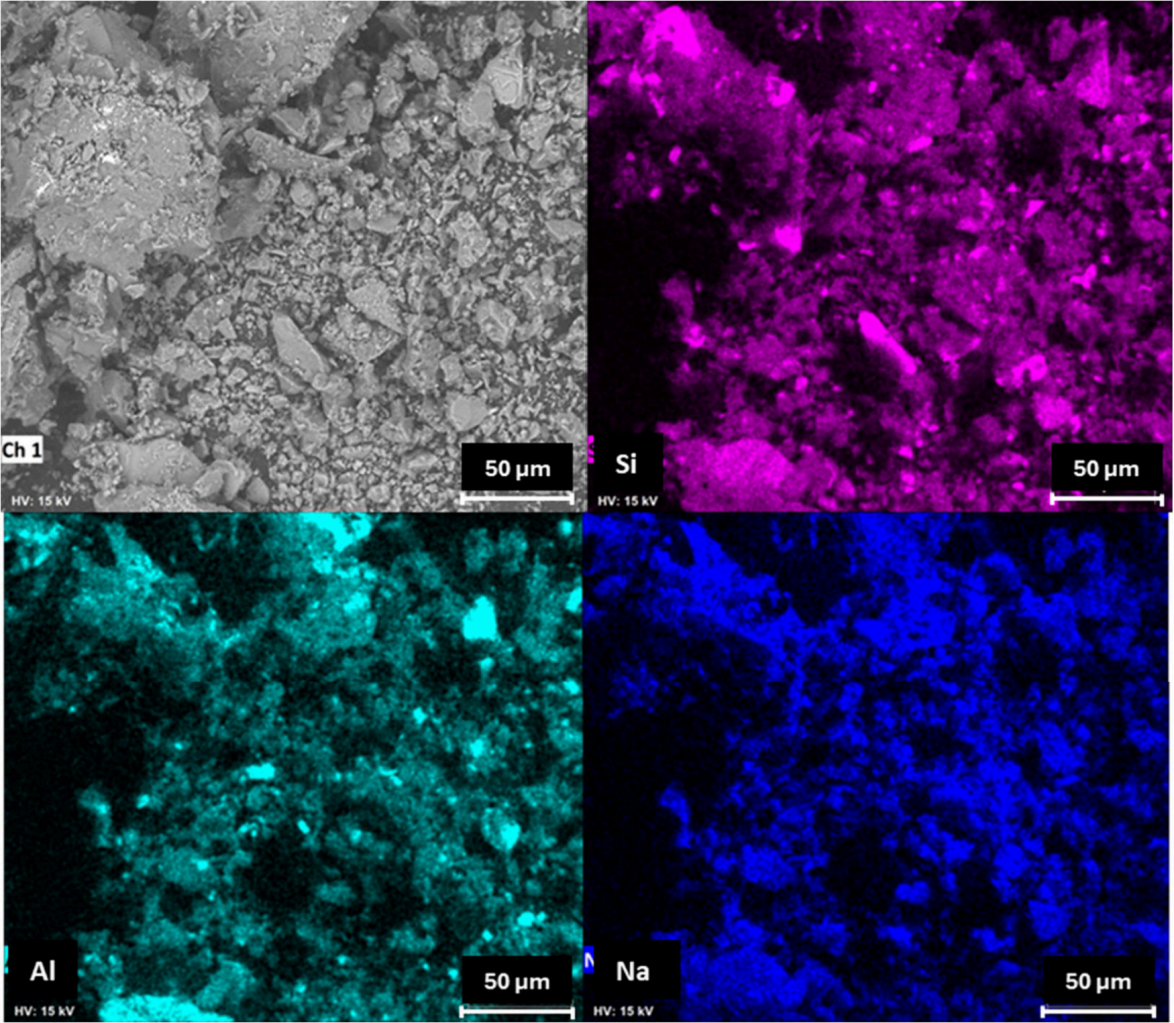
SEM image and elemental mapping of the geopolymer produced with soda-lime glass.

However, isolated particles rich in Si and Al, not coincident with Na, are observed in all geopolymer samples. These particles are likely residual quartz from the metakaolin, as previously identified in the X-ray diffraction (XRD) analyses. Quartz is a crystalline, nonreactive phase that remains after alkaline activation and does not participate in the formation of the geopolymeric network.^13^ The presence of such unreacted particles is commonly reported in metakaolin-based geopolymers. For instance, Jin et al. observed nonreactive particles in metakaolin geopolymers exhibiting heterogeneous microstructures with particles of variable sizes.^55^ Similarly, Khan et al. reported unreacted phases in advanced clay-based geopolymers, also displaying heterogeneous microstructures.^56^ These observations indicate that the microstructural heterogeneity of the geopolymers can be attributed to the presence of nonreactive phases such as residual quartz from the metakaolin.

The analyses performed on geopolymers synthesized from borosilicate and soda-lime glass waste demonstrated that the materials were successfully obtained, exhibiting the characteristic amorphous structure of aluminosilicate networks and chemical composition consistent with conventional geopolymers. Compressive strength tests, together with XRF, XRD, FTIR, and SEM/EDS characterizations, confirmed that the partial replacement of conventional precursors with up to 14% glass resulted in geopolymers with compressive strength comparable to or exceeding that of the standard matrix.

The results indicated that borosilicate glass, likely due to its higher Al_2_O_3_ content and smaller, more uniformly distributed particles, promoted the formation of a denser three-dimensional network, leading to higher compressive strength values. Soda-lime glass, although containing a lower aluminum content, also contributed effectively to network consolidation, demonstrating high reactivity under ambient curing conditions.

Comparison with the literature shows that the results are consistent. Table 4 presents a comparison of glass-based and other precursor-based geopolymers, illustrating that the compressive strength values obtained in this study fall within the range reported in previous works, even when considering more severe curing conditions, such as elevated temperatures (60–85 °C) or 28 day curing periods.

**Table 4:** Comparison of Compressive Strength Results, Curing Conditions, and Glass Substitutions from the Present Study and Relevant Literature, Highlighting the Effects of Different Types of Glass and Replacement Levels on Geopolymer Performance

Study	Type of glass/replacement	Substituted precursor	Curing temperature (°C)	Compressive strength (MPa)/curing time (days)
Cendrowski et al., 2025 ^28^	Ground lab glass/0, 10, 20, 30%	Fly ash	60 °C	17.0, 41.7, 32.6, 19.1/28 days
Kotsay and Grabowski, 2023 ^29^	Soda-lime glass/10, 30, 50%	Metakaolin	20 °C	32, 31, 34, 16/7 days
Tho-In et al., 2018 ^19^	Fluorescent lamp (FP)/10, 20, 30, 40%	Fly ash	60 °C	FP: 41.1, 40.7, 39.6, 33.3
	Container glass (CP)/10, 20, 30, 40%			CP: 44.4, 47.6, 39.9, 39.9/7 days
Torres-Carrasco and Puertas, 2015 ^20^	Urban waste glass/NaOH 10 M + 10 g glass, NaOH 10 M + 15 g glass, NaOH 10 M + 25 g glass	Sodium silicate	85 °C	32, 36, 14/7 days
This work	Borosilicate glass (BSG)/14, 17.4, 21.5, 25%	Sodium silicate and metakaolin	Room temperature: ∼25 °C	BSG: 33, 29, 27, 22
	Soda-lime glass (SLG)/14, 17.4, 21.5, 25%			SLG: 30, 24, 23, 18/7 days

A key highlight of this study is that compressive strengths exceeding 30 MPa were achieved after only 7 days of curing at room temperature without additional heating. This demonstrates the high reactivity of the glass powders and the efficiency of the adopted alkaline activation conditions. Furthermore, incorporating glass waste contributes to process sustainability by partially replacing metakaolin and sodium silicate, reducing costs and minimizing the environmental impact associated with geopolymer production.

In summary, the results confirm that both borosilicate and soda-lime glass waste can serve as viable precursors for high-performance geopolymers, providing an economically efficient and environmentally sustainable alternative for construction material manufacturing.

## Conclusions

This study demonstrated the successful production of geopolymers using borosilicate and soda-lime glass waste as partial substitutes for conventional raw materials. The characterization of the glasses indicated high silica content and predominantly amorphous structures, confirming their suitability as precursors for geopolymer synthesis. Geopolymers were produced with different levels of glass incorporation, and mechanical tests performed after 7 days of curing revealed that for soda-lime glass, the optimal compressive strength was obtained with 14% incorporation (∼30 MPa), while higher contents led to a gradual decrease. For borosilicate glass, geopolymers with 14% and up to 21.5% incorporation exhibited compressive strengths comparable to or higher than that of the conventional matrix (∼27–32 MPa), demonstrating that higher amounts of this glass can be effectively used. Notably, these formulations enabled a substantial reduction in sodium silicate usage, a high-cost reagent, which represents a relevant economic advantage. The mechanical tests achieved suggest that glass-based geopolymers may be considered as potential alternatives for applications where compressive strengths comparable to that of ordinary Portland cement are required. Structural and morphological analyses (XRF, XRD, FTIR, and SEM/EDS) confirmed the formation of geopolymeric networks and were consistent with the mechanical results. Overall, the use of glass waste contributes to material valorization and supports the development of more sustainable binder systems with reduced environmental impact.

## Supplementary Material



## Notes

Additional photograph of the glass samples used as raw materials and image of the geopolymer specimens prior to demolding (PDF)
