# Supplementary material for: Soda-Lime and Borosilicate Glass Waste as Precursors for Sustainable Geopolymers
Source: ACS Omega. 2026 Apr 16;11(16):23976–86. doi: 10.1021/acsomega.5c11890 (PMC13130126; doi:10.1021/acsomega.5c11890)
Supplement: Supplementary file 1 [file ao5c11890_si_001.pdf]

SUPLEMENTARY MATERIAL

Soda-Lime and Borosilicate Glass Waste as Precursors for Sustainable Geopolymers

*Caroline D. Prates[1], Wladmir T. Silva[1], Athos Silva Lima[1], Ana Gabriela D. Santana[1], Luádiny V. L. Santos[1], Ana Paula C. Teixeira[1]**

[1]UFMG, Departamento de Química/Icex, Belo Horizonte, Minas Gerais, Brazil, 31270-901

*e-mail: anapct@ufmg.br

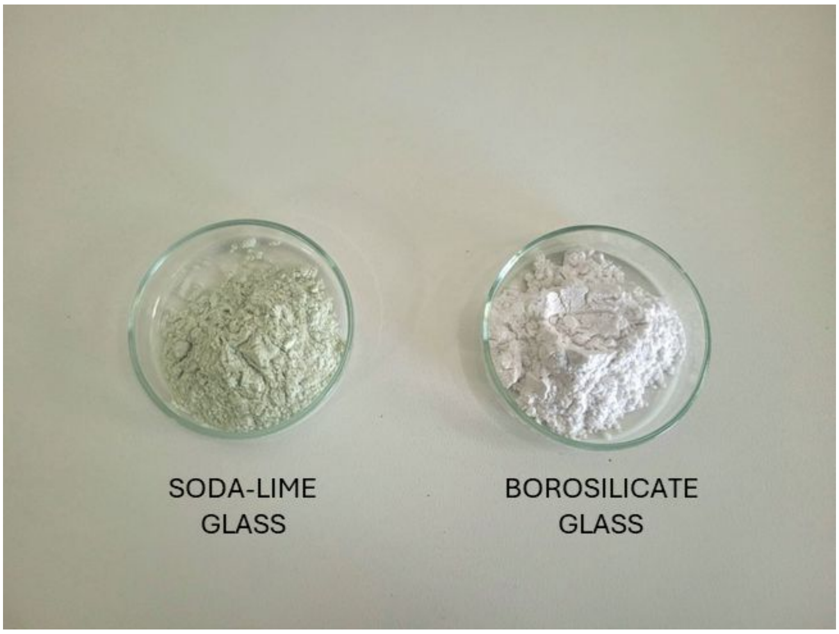

Figure S1. Photos of samples of glass waste used

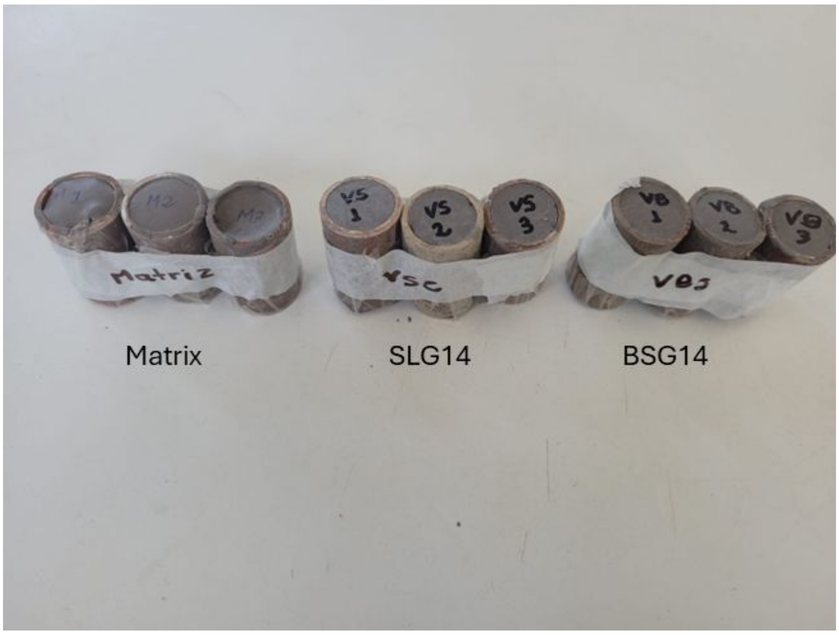

Figure S2. Geopolymers matrix (without glass addition), SLG14 (with 14% soda-lime glass), and BSG14 (with 14% borosilicate glass) before demolding
